# Chloridobis(1,10-phenanthroline-κ^2^ *N*,*N*′)(2,2,2-trichloro­acetato-κ*O*)cobalt(II)

**DOI:** 10.1107/S1600536809054671

**Published:** 2009-12-24

**Authors:** Changgui Pei, Peikang Bai, Zhangxia Guo

**Affiliations:** a School of Electronics and Computer Science and Technology, North University of China, Taiyuan 030051, People’s Republic of China; b School of Materials Science and Engineering, North University of China, Taiyuan 030051, People’s Republic of China; c School of Electronic Science and Technology, North University of China, Taiyuan 030051, People’s Republic of China

## Abstract

The title compound, [Co(C_2_Cl_3_O_2_)Cl(C_12_H_8_N_2_)_2_], was obtained by the reaction of trichloro­acetic acid and CoCl_2_ in the presence of 1,10-phenanthroline. The Co^II^ ion exhibits a distorted octa­hedral geometry, with three N atoms from two 1,10-phenanthroline ligands and the Cl^−^ ion in the equatorial plane and one O atom from the trichloro­acetate ligand and one phenanthroline N atom in axial positions. This compound is isostructural with the analogous Mn^II^ complex. The trichloro­methyl group of the trichloro­acetate ligand is disordered over two positions with occupancies of 0.190 (5) and 0.810 (5).

## Related literature

For the structure of isostructural Mn^II ^complex, see: Chen *et al.* (2006 ▶).
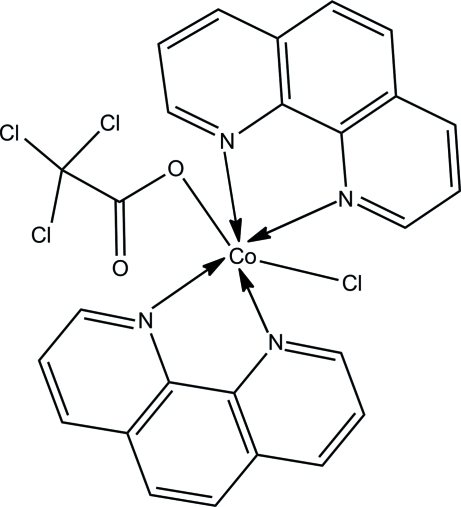


## Experimental

### 

#### Crystal data

[Co(C_2_Cl_3_O_2_)Cl(C_12_H_8_N_2_)_2_]
*M* *_r_* = 617.16
Monoclinic, 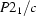

*a* = 18.2170 (6) Å
*b* = 10.4612 (4) Å
*c* = 14.6638 (7) Å
β = 112.685 (1)°
*V* = 2578.32 (18) Å^3^
*Z* = 4
Mo *K*α radiation
μ = 1.12 mm^−1^
*T* = 293 K
0.26 × 0.20 × 0.18 mm

#### Data collection

Bruker SMART APEX diffractometer
Absorption correction: multi-scan (*SADABS*; Sheldrick, 2005 ▶) *T*_min_ = 0.760, *T*_max_ = 0.824
13155 measured reflections
4536 independent reflections
3821 reflections with *I* > 2σ(*I*)
*R*_int_ = 0.047

#### Refinement

*R*[*F* ^2^ > 2σ(*F* ^2^)] = 0.031
*wR*(*F* ^2^) = 0.086
*S* = 1.01
4536 reflections
344 parameters
H-atom parameters constrained
Δρ_max_ = 0.56 e Å^−3^
Δρ_min_ = −0.62 e Å^−3^

### 

Data collection: *SMART* (Bruker, 2005 ▶); cell refinement: *SAINT* (Bruker, 2005 ▶); data reduction: *SAINT*; program(s) used to solve structure: *SHELXS97* (Sheldrick, 2008 ▶); program(s) used to refine structure: *SHELXL97* (Sheldrick, 2008 ▶); molecular graphics: *SHELXTL* (Sheldrick, 2008 ▶); software used to prepare material for publication: *SHELXL97*.

## Supplementary Material

Crystal structure: contains datablocks I, global. DOI: 10.1107/S1600536809054671/gk2250sup1.cif

Structure factors: contains datablocks I. DOI: 10.1107/S1600536809054671/gk2250Isup2.hkl

Additional supplementary materials:  crystallographic information; 3D view; checkCIF report

## Figures and Tables

**Table 1:** Selected bond lengths (Å)

Co1—O2	2.078 (2)
Co1—N4	2.155 (2)
Co1—N3	2.161 (2)
Co1—N2	2.172 (2)
Co1—N1	2.190 (2)
Co1—Cl4	2.3985 (6)

**Fig. 1.**
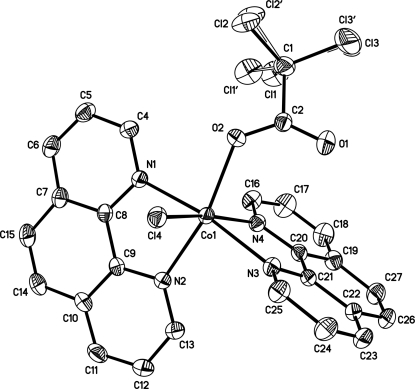
The molecular structure of title compound, with atom labels and 30% probability displacement ellipsoids for non-H atoms.

## supplementary crystallographic information

### Comment

The molecular structure of the title compound is shown in Fig. 1. The Co atom exhibits a distorted octahedral geometry. The metal ion deviates from the plane defined by three N atoms from two phenantroline molecules and the chlorido ligand by 0.0881 (2) Å.

### Experimental

The reaction was carried out by the hydrothermal method. Trichloroacetic acid (0.082 g, 0.5 mmol), CoCl_2_.6H_2_O_._(0.119 g, 0.5 mmol) and 1,10-phenanthroline (0.180 g, 1 mmol) were added to the airtight vessel containing 20 ml of water/methanol mixture in 2:1 ratio. The reaction was carried out at 303 K for 4 days and than cooled down. Resulting brown solution was filtered and brown block-shaped crystals appeared within a few days. Yield 76%; analysis calc. for C_26_H_16_Cl_4_CoN_4_O_2_: C 50.60, H 2.61, N 9.08%; found: C 50.91, H 2.25, N 9.34%. The elemental analyses were performed with PERKIN ELMER Model 2400 Series II.

### Refinement

H atoms were positioned geometrically and treated as riding with with C—H = 0.93 Å and U_iso_(H) = 1.2Ue_q_(C). The trichloromethyl group is disordered. Occupancies of Cl atoms in two positions refined at 0.190 (5) and 0.810 (5).. No restraints were imposed on the geometry of the disordered group.

### Crystal data

**Table:**

[Co(C_2_Cl_3_O_2_)Cl(C_12_H_8_N_2_)_2_]	*F*(000) = 1244
*M**_r_* = 617.16	*D*_x_ = 1.595 Mg m^−^^3^
Monoclinic, *P*2_1_/*c*	Mo *K*α radiation, λ = 0.71073 Å
Hall symbol: -P 2ybc	Cell parameters from 7284 reflections
*a* = 18.2170 (6) Å	θ = 2.3–28.1°
*b* = 10.4612 (4) Å	µ = 1.12 mm^−^^1^
*c* = 14.6638 (7) Å	*T* = 293 K
β = 112.685 (1)°	Block, brown
*V* = 2578.32 (18) Å^3^	0.26 × 0.20 × 0.18 mm
*Z* = 4	

### Data collection

**Table:**

Bruker SMART APEX diffractometer	4536 independent reflections
Radiation source: fine-focus sealed tube	3821 reflections with *I* > 2σ(*I*)
graphite	*R*_int_ = 0.047
Detector resolution: 0 pixels mm^-1^	θ_max_ = 25.1°, θ_min_ = 2.3°
phi and ω scans	*h* = −17→21
Absorption correction: multi-scan (*SADABS*; Sheldrick, 2005)	*k* = −11→12
*T*_min_ = 0.760, *T*_max_ = 0.824	*l* = −17→16
13155 measured reflections	

### Refinement

**Table:**

Refinement on *F*^2^	Primary atom site location: structure-invariant direct methods
Least-squares matrix: full	Secondary atom site location: difference Fourier map
*R*[*F*^2^ > 2σ(*F*^2^)] = 0.031	Hydrogen site location: inferred from neighbouring sites
*wR*(*F*^2^) = 0.086	H-atom parameters constrained
*S* = 1.01	*w* = 1/[σ^2^(*F*_o_^2^) + (0.042*P*)^2^ + 0.9036*P*] where *P* = (*F*_o_^2^ + 2*F*_c_^2^)/3
4536 reflections	(Δ/σ)_max_ = 0.001
344 parameters	Δρ_max_ = 0.56 e Å^−^^3^
0 restraints	Δρ_min_ = −0.62 e Å^−^^3^

### Special details

**Table:**

Geometry. All e.s.d.'s (except the e.s.d. in the dihedral angle between two l.s. planes) are estimated using the full covariance matrix. The cell e.s.d.'s are taken into account individually in the estimation of e.s.d.'s in distances, angles and torsion angles; correlations between e.s.d.'s in cell parameters are only used when they are defined by crystal symmetry. An approximate (isotropic) treatment of cell e.s.d.'s is used for estimating e.s.d.'s involving l.s. planes.
Refinement. Refinement of *F*^2^ against ALL reflections. The weighted *R*-factor *wR* and goodness of fit *S* are based on *F*^2^, conventional *R*-factors *R* are based on *F*, with *F* set to zero for negative *F*^2^. The threshold expression of *F*^2^ > σ(*F*^2^) is used only for calculating *R*-factors(gt) *etc*. and is not relevant to the choice of reflections for refinement. *R*-factors based on *F*^2^ are statistically about twice as large as those based on *F*, and *R*- factors based on ALL data will be even larger.

### Fractional atomic coordinates and isotropic or equivalent isotropic displacement parameters (Å^2^)

**Table:**

	*x*	*y*	*z*	*U*_iso_*/*U*_eq_	Occ. (<1)
Co1	0.290424 (16)	0.88315 (3)	0.12391 (2)	0.03091 (10)	
N1	0.39845 (10)	0.79092 (17)	0.12432 (13)	0.0340 (4)	
N2	0.36251 (10)	1.04002 (17)	0.10756 (13)	0.0360 (4)	
N3	0.19137 (10)	1.00424 (18)	0.11353 (13)	0.0347 (4)	
N4	0.22255 (11)	0.89736 (17)	−0.03298 (13)	0.0349 (4)	
O1	0.11352 (10)	0.7113 (2)	0.04032 (17)	0.0690 (6)	
O2	0.24513 (9)	0.69856 (14)	0.10847 (11)	0.0389 (4)	
Cl1	0.1960 (6)	0.5139 (6)	−0.0763 (5)	0.0730 (5)	0.190 (5)
Cl2	0.229 (3)	0.430 (3)	0.132 (4)	0.0645 (8)	0.190 (5)
Cl3	0.0807 (13)	0.462 (2)	−0.0130 (11)	0.1180 (11)	0.190 (5)
Cl1'	0.23758 (16)	0.50950 (12)	−0.04800 (13)	0.0730 (5)	0.810 (5)
Cl2'	0.2220 (6)	0.4154 (7)	0.1267 (8)	0.0645 (8)	0.810 (5)
Cl3'	0.0798 (3)	0.4598 (4)	−0.0455 (2)	0.1180 (11)	0.810 (5)
Cl4	0.34432 (3)	0.89244 (5)	0.30104 (4)	0.04054 (15)	
C1	0.17636 (16)	0.5183 (2)	0.02503 (19)	0.0511 (6)	
C2	0.17667 (13)	0.6599 (2)	0.06043 (16)	0.0364 (5)	
C4	0.41513 (14)	0.6677 (2)	0.13065 (18)	0.0424 (5)	
H4	0.3759	0.6100	0.1294	0.051*	
C5	0.48902 (15)	0.6199 (2)	0.13921 (19)	0.0500 (6)	
H5	0.4980	0.5322	0.1423	0.060*	
C6	0.54786 (14)	0.7022 (3)	0.14301 (19)	0.0491 (6)	
H6	0.5975	0.6712	0.1496	0.059*	
C7	0.53335 (13)	0.8331 (2)	0.13705 (16)	0.0395 (5)	
C8	0.45653 (12)	0.8735 (2)	0.12710 (15)	0.0325 (5)	
C9	0.43734 (12)	1.0069 (2)	0.11826 (15)	0.0327 (5)	
C10	0.49480 (14)	1.0973 (2)	0.11934 (17)	0.0405 (5)	
C11	0.47207 (15)	1.2252 (2)	0.10689 (19)	0.0505 (6)	
H11	0.5082	1.2882	0.1071	0.061*	
C12	0.39604 (16)	1.2573 (2)	0.0943 (2)	0.0547 (7)	
H12	0.3797	1.3422	0.0850	0.066*	
C13	0.34353 (14)	1.1621 (2)	0.09570 (19)	0.0467 (6)	
H13	0.2922	1.1856	0.0878	0.056*	
C14	0.57281 (14)	1.0536 (3)	0.13301 (19)	0.0498 (6)	
H14	0.6118	1.1131	0.1368	0.060*	
C15	0.59105 (14)	0.9289 (3)	0.14048 (19)	0.0506 (6)	
H15	0.6420	0.9037	0.1480	0.061*	
C16	0.23604 (15)	0.8363 (3)	−0.10371 (17)	0.0446 (6)	
H16	0.2808	0.7844	−0.0865	0.054*	
C17	0.18590 (17)	0.8463 (3)	−0.20312 (18)	0.0590 (7)	
H17	0.1963	0.7997	−0.2508	0.071*	
C18	0.12179 (17)	0.9246 (3)	−0.22945 (19)	0.0578 (7)	
H18	0.0890	0.9340	−0.2958	0.069*	
C19	0.10473 (14)	0.9916 (2)	−0.15691 (17)	0.0431 (5)	
C20	0.15708 (12)	0.9720 (2)	−0.05844 (16)	0.0344 (5)	
C21	0.14063 (12)	1.0312 (2)	0.01997 (16)	0.0328 (5)	
C22	0.07409 (13)	1.1109 (2)	−0.00170 (18)	0.0389 (5)	
C23	0.06112 (14)	1.1647 (2)	0.0780 (2)	0.0465 (6)	
H23	0.0177	1.2182	0.0671	0.056*	
C24	0.11226 (15)	1.1384 (3)	0.1715 (2)	0.0498 (6)	
H24	0.1044	1.1741	0.2251	0.060*	
C25	0.17703 (15)	1.0572 (2)	0.18682 (17)	0.0440 (6)	
H25	0.2115	1.0397	0.2514	0.053*	
C26	0.02322 (14)	1.1322 (2)	−0.1033 (2)	0.0487 (6)	
H26	−0.0207	1.1857	−0.1185	0.058*	
C27	0.03816 (15)	1.0759 (3)	−0.17682 (19)	0.0509 (6)	
H27	0.0046	1.0919	−0.2420	0.061*	

### Atomic displacement parameters (Å^2^)

**Table:**

	*U*^11^	*U*^22^	*U*^33^	*U*^12^	*U*^13^	*U*^23^
Co1	0.02742 (16)	0.03019 (17)	0.03545 (17)	0.00155 (11)	0.01248 (13)	0.00274 (12)
N1	0.0299 (9)	0.0333 (10)	0.0382 (10)	0.0020 (8)	0.0124 (8)	0.0025 (8)
N2	0.0326 (10)	0.0319 (10)	0.0427 (10)	−0.0007 (8)	0.0135 (8)	0.0015 (8)
N3	0.0319 (9)	0.0373 (10)	0.0358 (9)	0.0029 (8)	0.0141 (8)	0.0018 (8)
N4	0.0331 (9)	0.0361 (10)	0.0379 (10)	0.0018 (8)	0.0165 (8)	0.0033 (8)
O1	0.0364 (10)	0.0618 (12)	0.1007 (16)	0.0080 (9)	0.0175 (10)	−0.0022 (12)
O2	0.0374 (9)	0.0346 (8)	0.0444 (9)	−0.0048 (7)	0.0153 (7)	0.0011 (7)
Cl1	0.0975 (14)	0.0771 (6)	0.0503 (7)	0.0091 (8)	0.0352 (9)	−0.0132 (5)
Cl2	0.085 (2)	0.039 (2)	0.0671 (15)	0.0147 (14)	0.0268 (15)	0.0094 (15)
Cl3	0.0788 (7)	0.0729 (7)	0.132 (2)	−0.0317 (5)	−0.0366 (18)	−0.0075 (18)
Cl1'	0.0975 (14)	0.0771 (6)	0.0503 (7)	0.0091 (8)	0.0352 (9)	−0.0132 (5)
Cl2'	0.085 (2)	0.039 (2)	0.0671 (15)	0.0147 (14)	0.0268 (15)	0.0094 (15)
Cl3'	0.0788 (7)	0.0729 (7)	0.132 (2)	−0.0317 (5)	−0.0366 (18)	−0.0075 (18)
Cl4	0.0363 (3)	0.0438 (3)	0.0378 (3)	0.0023 (2)	0.0102 (2)	0.0029 (2)
C1	0.0529 (15)	0.0415 (14)	0.0488 (14)	−0.0056 (12)	0.0083 (12)	−0.0031 (12)
C2	0.0328 (12)	0.0406 (12)	0.0364 (11)	0.0010 (10)	0.0141 (10)	0.0060 (10)
C4	0.0405 (13)	0.0347 (12)	0.0517 (14)	0.0037 (10)	0.0175 (11)	0.0037 (11)
C5	0.0478 (15)	0.0431 (14)	0.0580 (16)	0.0132 (12)	0.0191 (13)	0.0008 (12)
C6	0.0373 (13)	0.0576 (16)	0.0536 (15)	0.0134 (12)	0.0187 (11)	−0.0004 (13)
C7	0.0326 (12)	0.0509 (14)	0.0351 (12)	0.0039 (11)	0.0132 (10)	−0.0011 (11)
C8	0.0291 (11)	0.0394 (12)	0.0281 (10)	−0.0001 (9)	0.0101 (9)	0.0000 (9)
C9	0.0296 (11)	0.0386 (12)	0.0288 (10)	−0.0033 (9)	0.0100 (9)	−0.0014 (9)
C10	0.0383 (13)	0.0444 (14)	0.0375 (12)	−0.0103 (10)	0.0131 (10)	−0.0036 (10)
C11	0.0487 (15)	0.0430 (15)	0.0584 (15)	−0.0164 (12)	0.0190 (13)	−0.0025 (12)
C12	0.0532 (15)	0.0335 (13)	0.0740 (18)	−0.0048 (11)	0.0207 (14)	0.0020 (13)
C13	0.0406 (13)	0.0348 (13)	0.0637 (16)	0.0012 (11)	0.0189 (12)	0.0033 (12)
C14	0.0342 (13)	0.0600 (17)	0.0561 (15)	−0.0138 (12)	0.0185 (11)	−0.0052 (13)
C15	0.0294 (12)	0.0687 (18)	0.0535 (15)	−0.0018 (12)	0.0159 (11)	−0.0054 (14)
C16	0.0477 (13)	0.0512 (14)	0.0424 (13)	0.0060 (12)	0.0257 (11)	0.0028 (12)
C17	0.0686 (18)	0.077 (2)	0.0373 (13)	0.0120 (16)	0.0266 (13)	−0.0001 (13)
C18	0.0593 (17)	0.0757 (19)	0.0336 (13)	0.0056 (15)	0.0126 (12)	0.0058 (13)
C19	0.0401 (13)	0.0494 (14)	0.0367 (12)	−0.0021 (11)	0.0114 (10)	0.0057 (11)
C20	0.0304 (11)	0.0355 (12)	0.0370 (11)	−0.0028 (9)	0.0128 (9)	0.0039 (10)
C21	0.0270 (11)	0.0298 (11)	0.0405 (12)	−0.0017 (9)	0.0118 (9)	0.0034 (9)
C22	0.0293 (11)	0.0332 (12)	0.0515 (14)	−0.0003 (9)	0.0128 (10)	0.0035 (10)
C23	0.0375 (13)	0.0414 (13)	0.0639 (16)	0.0083 (11)	0.0233 (12)	0.0014 (12)
C24	0.0509 (15)	0.0520 (15)	0.0535 (15)	0.0090 (12)	0.0278 (13)	−0.0039 (13)
C25	0.0454 (13)	0.0492 (14)	0.0395 (12)	0.0066 (11)	0.0186 (11)	−0.0003 (11)
C26	0.0337 (12)	0.0450 (14)	0.0580 (16)	0.0089 (11)	0.0073 (11)	0.0098 (12)
C27	0.0426 (14)	0.0561 (16)	0.0428 (13)	0.0062 (12)	0.0040 (11)	0.0097 (13)

### Geometric parameters (Å, °)

**Table:**

Co1—O2	2.078 (2)	C9—C10	1.406 (3)
Co1—N4	2.155 (2)	C10—C11	1.392 (4)
Co1—N3	2.161 (2)	C10—C14	1.431 (3)
Co1—N2	2.172 (2)	C11—C12	1.367 (4)
Co1—N1	2.190 (2)	C11—H11	0.9300
Co1—Cl4	2.3985 (6)	C12—C13	1.386 (3)
N1—C4	1.320 (3)	C12—H12	0.9300
N1—C8	1.355 (3)	C13—H13	0.9300
N2—C13	1.316 (3)	C14—C15	1.340 (4)
N2—C9	1.356 (3)	C14—H14	0.9300
N3—C25	1.321 (3)	C15—H15	0.9300
N3—C21	1.356 (3)	C16—C17	1.394 (3)
N4—C16	1.320 (3)	C16—H16	0.9300
N4—C20	1.352 (3)	C17—C18	1.355 (4)
O1—C2	1.199 (3)	C17—H17	0.9300
O2—C2	1.240 (3)	C18—C19	1.405 (4)
Cl1—C1	1.658 (6)	C18—H18	0.9300
Cl2—C1	1.75 (5)	C19—C20	1.405 (3)
Cl3—C1	1.72 (2)	C19—C27	1.435 (4)
Cl1'—C1	1.822 (3)	C20—C21	1.436 (3)
Cl2'—C1	1.766 (11)	C21—C22	1.403 (3)
Cl3'—C1	1.770 (4)	C22—C23	1.398 (3)
C1—C2	1.569 (3)	C22—C26	1.436 (3)
C4—C5	1.395 (3)	C23—C24	1.355 (4)
C4—H4	0.9300	C23—H23	0.9300
C5—C6	1.359 (4)	C24—C25	1.400 (3)
C5—H5	0.9300	C24—H24	0.9300
C6—C7	1.390 (4)	C25—H25	0.9300
C6—H6	0.9300	C26—C27	1.346 (4)
C7—C8	1.415 (3)	C26—H26	0.9300
C7—C15	1.439 (3)	C27—H27	0.9300
C8—C9	1.432 (3)		
			
O2—Co1—N4	84.70 (6)	N2—C9—C10	122.7 (2)
O2—Co1—N3	104.56 (6)	N2—C9—C8	117.34 (19)
N4—Co1—N3	76.40 (7)	C10—C9—C8	120.0 (2)
O2—Co1—N2	157.18 (6)	C11—C10—C9	117.6 (2)
N4—Co1—N2	87.28 (7)	C11—C10—C14	123.7 (2)
N3—Co1—N2	94.21 (7)	C9—C10—C14	118.8 (2)
O2—Co1—N1	84.80 (6)	C12—C11—C10	119.2 (2)
N4—Co1—N1	100.27 (7)	C12—C11—H11	120.4
N3—Co1—N1	169.56 (7)	C10—C11—H11	120.4
N2—Co1—N1	75.65 (7)	C11—C12—C13	119.4 (2)
O2—Co1—Cl4	97.77 (5)	C11—C12—H12	120.3
N4—Co1—Cl4	168.35 (5)	C13—C12—H12	120.3
N3—Co1—Cl4	91.98 (5)	N2—C13—C12	123.4 (2)
N2—Co1—Cl4	94.39 (5)	N2—C13—H13	118.3
N1—Co1—Cl4	91.31 (5)	C12—C13—H13	118.3
C4—N1—C8	117.74 (19)	C15—C14—C10	121.6 (2)
C4—N1—Co1	127.80 (15)	C15—C14—H14	119.2
C8—N1—Co1	114.18 (14)	C10—C14—H14	119.2
C13—N2—C9	117.68 (19)	C14—C15—C7	121.3 (2)
C13—N2—Co1	127.44 (15)	C14—C15—H15	119.3
C9—N2—Co1	114.68 (14)	C7—C15—H15	119.3
C25—N3—C21	117.66 (19)	N4—C16—C17	122.7 (2)
C25—N3—Co1	127.58 (15)	N4—C16—H16	118.7
C21—N3—Co1	114.71 (14)	C17—C16—H16	118.7
C16—N4—C20	118.3 (2)	C18—C17—C16	119.2 (2)
C16—N4—Co1	126.95 (16)	C18—C17—H17	120.4
C20—N4—Co1	114.59 (14)	C16—C17—H17	120.4
C2—O2—Co1	129.44 (15)	C17—C18—C19	120.3 (2)
C2—C1—Cl1	110.4 (3)	C17—C18—H18	119.9
C2—C1—Cl3	107.9 (8)	C19—C18—H18	119.9
Cl1—C1—Cl3	104.0 (7)	C20—C19—C18	116.4 (2)
C2—C1—Cl2	105.5 (12)	C20—C19—C27	119.0 (2)
Cl1—C1—Cl2	123.8 (18)	C18—C19—C27	124.7 (2)
Cl3—C1—Cl2	104.3 (15)	N4—C20—C19	123.0 (2)
C2—C1—Cl2'	110.8 (3)	N4—C20—C21	117.54 (19)
C2—C1—Cl3'	113.3 (2)	C19—C20—C21	119.4 (2)
Cl2'—C1—Cl3'	108.8 (3)	N3—C21—C22	123.0 (2)
C2—C1—Cl1'	108.47 (17)	N3—C21—C20	116.70 (18)
Cl2'—C1—Cl1'	105.7 (4)	C22—C21—C20	120.3 (2)
Cl3'—C1—Cl1'	109.5 (2)	C23—C22—C21	117.4 (2)
O1—C2—O2	130.7 (2)	C23—C22—C26	123.7 (2)
O1—C2—C1	117.5 (2)	C21—C22—C26	118.9 (2)
O2—C2—C1	111.7 (2)	C24—C23—C22	119.5 (2)
N1—C4—C5	123.0 (2)	C24—C23—H23	120.3
N1—C4—H4	118.5	C22—C23—H23	120.3
C5—C4—H4	118.5	C23—C24—C25	119.6 (2)
C6—C5—C4	119.7 (2)	C23—C24—H24	120.2
C6—C5—H5	120.2	C25—C24—H24	120.2
C4—C5—H5	120.2	N3—C25—C24	122.9 (2)
C5—C6—C7	119.6 (2)	N3—C25—H25	118.6
C5—C6—H6	120.2	C24—C25—H25	118.6
C7—C6—H6	120.2	C27—C26—C22	120.9 (2)
C6—C7—C8	117.2 (2)	C27—C26—H26	119.5
C6—C7—C15	124.4 (2)	C22—C26—H26	119.5
C8—C7—C15	118.4 (2)	C26—C27—C19	121.5 (2)
N1—C8—C7	122.8 (2)	C26—C27—H27	119.3
N1—C8—C9	117.28 (18)	C19—C27—H27	119.3
C7—C8—C9	119.9 (2)		

### Hydrogen-bond geometry (Å, °)

**Table:**

*D*—H···*A*	*D*—H	H···*A*	*D*···*A*	*D*—H···*A*
C17—H17···O2^i^	0.93	2.54	3.364 (3)	148
C11—H11···Cl4^ii^	0.93	2.73	3.548 (2)	148
C23—H23···O1^iii^	0.93	2.42	3.249 (3)	149

Symmetry codes: (i) *x*, −*y*+3/2, *z*−1/2; (ii) −*x*+1, *y*+1/2, −*z*+1/2; (iii) −*x*, −*y*+2, −*z*.
